# Young people’s experiences of receiving therapist-supported online cognitive therapy for post-traumatic stress disorder

**DOI:** 10.1080/20008066.2026.2744046

**Published:** 2026-11-02

**Authors:** Helena Griffiths, Richard Meiser-Stedman, Ewan Carr, David Clark, Tim Dalgleish, Anke Ehlers, Kimberly Goldsmith, Monica Gupta, Dorothy King, Sarah Miles, Dominic Plant, Anne Smith, Jessica Steward, Patrick Smith

**Affiliations:** a Department of Psychology, Institute of Psychiatry, Psychology and Neuroscience, King’s College London, London, UK; b South London & Maudsley NHS Foundation Trust, London, UK; c Department of Clinical Psychology and Psychological Therapies, University of East Anglia, Norwich, UK; d Department of Biostatistics and Health Informatics, King’s College London, London, UK; e Department of Experimental Psychology, University of Oxford, Oxford, UK; f MRC Cognition and Brain Sciences Unit, University of Cambridge, Cambridge, UK; g Department of Psychology, University of Cambridge, Cambridge, UK; h Berkshire Healthcare NHS Foundation Trust, Reading, Berkshire, UK; i Norfolk and Suffolk NHS Foundation Trust, Norwich, UK

**Keywords:** Post-traumatic stress disorder, PTSD, young people, digital, cognitive therapy, qualitative analysis, internet and mobile-based intervention, Trastorno de estrés postraumático, TEPT, jóvenes, digital, terapia cognitiva, análisis cualitativo, intervención basada en internet y dispositivos móviles

## Abstract

**Background:** Effective face-to-face treatments for post-traumatic stress disorder (PTSD) exist, but most young people with PTSD are not offered evidence-based treatment. Internet-delivered cognitive therapy for post-traumatic stress disorder in young people (iCT-PTSD-YP) may increase access to effective treatment.

**Objective:** This qualitative study aimed to explore the experiences of young people receiving iCT-PTSD-YP.

**Method:** Semi-structured interviews were undertaken with 20 young people, aged 12–17 years, who had participated in an early-stage randomized controlled trial (RCT). Reflexive thematic analysis was used to identify key themes and subthemes.

**Results:** Three overarching themes were identified: ‘Accessibility’, ‘Engagement’, and ‘Expectations and outcomes’. The therapist-supported digital therapy was perceived as accessible, available, and easy to use and navigate. iCT-PTSD-YP was found to be convenient and flexible, and taking part in an RCT offered the opportunity to access thorough assessment and treatment. The level of engagement depended on individual and contextual factors, and was helped by the support of a therapist, a well-designed digital platform with engaging therapeutic content, and receiving treatment in a safe and comfortable environment. iCT-PTSD-YP was perceived as efficacious by the majority of young people, but requiring a level of hard work, motivation, and commitment.

**Conclusions:** The findings highlight the potential utility of a therapist-supported digital intervention for PTSD in young people. Factors that may impact engagement and outcomes should be incorporated in future changes to design and delivery to improve acceptability and accessibility.

## HIGHLIGHTS

- Young people report that a therapist-supported digital mental health intervention for PTSD is broadly acceptable and viewed as accessible, flexible, and convenient.
- App-delivered memory-focused work is feasible and safe, and was perceived as helpful by young people.
- Therapist support was reported to be essential to engagement and overall treatment delivery.

## Abstract (es)

**Antecedentes:** Existen tratamientos presenciales eficaces para el trastorno de estrés postraumático (TEPT), pero a la mayoría de los jóvenes con TEPT no se les ofrece un tratamiento basado en la evidencia. La terapia cognitiva para el TEPT en jóvenes impartida a través de Internet (iCT-PTSD-YP, por su sigla en Inglés) podría aumentar el acceso a tratamientos eficaces.

**Objetivo:** Este estudio cualitativo tuvo como objetivo explorar las experiencias de los jóvenes que recibían iCT-PTSD-YP.

**Métodos:** Se realizaron entrevistas semiestructuradas a 20 jóvenes de entre 12 y 17 años que habían participado en un ensayo controlado aleatorizado (ECA) en fase inicial. Se utilizó el ‘Análisis Temático Reflexivo’ para identificar los temas y subtemas clave.

**Resultados:** Se identificaron tres temas generales: ‘accesibilidad’, ‘implicación’, y ‘expectativas y resultados’. La terapia digital con apoyo de un terapeuta se percibió como accesible, disponible y fácil de usar y de navegar; se consideró que iCT-PTSD-YP resultaba práctica y flexible, y que participar en un ensayo controlado aleatorizado (ECA) ofrecía la oportunidad de acceder a una evaluación y un tratamiento exhaustivos. El nivel de implicación dependió de factores individuales y contextuales, y se vio favorecido por el apoyo de un terapeuta, una plataforma digital bien diseñada con contenido terapéutico atractivo y la recepción del tratamiento en un entorno seguro y cómodo. La mayoría de los jóvenes percibió iCT-PTSD-YP como eficaz, aunque el programa requería cierto nivel de compromiso para lograr los mejores resultados.

**Conclusiones:** Los hallazgos destacan la utilidad potencial de una intervención digital con apoyo de un terapeuta para el TEPT en jóvenes. En futuras modificaciones del diseño y la implementación, deberían incorporarse factores que puedan influir en la participación y los resultados, con el fin de mejorar la aceptabilidad y la accesibilidad.

## 1. Introduction

Post-traumatic stress disorder (PTSD) in young people is highly distressing, is associated with personal and societal costs (McGowan, 2019), and results in significant impairment in functioning, which can persist into adulthood if left untreated (Yule et al., 2000).

Trauma-focused cognitive behavioural therapies (TF-CBTs) have shown consistently large effects in reducing PTSD symptoms (Hoppen et al., 2025), resulting in their being recommended as first-line intervention in national and international treatment guidelines (Forbes et al., 2020; National Institute for Health and Care Excellence [NICE], 2018). Cognitive therapy for post-traumatic stress disorder in young people (CT-PTSD-YP) (Smith et al., 2010) is a form of TF-CBT based on cognitive theory (Ehlers & Clark, 2000; Meiser-Stedman et al., 2019), and was adapted from CT-PTSD for adults (Ehlers et al., 2005). In-person CT-PTSD-YP was found to be an efficacious and acceptable treatment in three randomized controlled trials (RCTs) (Hitchcock et al., 2022; Meiser-Stedman et al., 2017; Smith et al., 2007).

However, most young people with PTSD do not receive evidence-based treatments (Lewis et al., 2019). This may be due to multiple factors, including the stigma associated with attending mental health services, UK mental health system capacity issues, living in areas with little or no access to specialist mental health services, lack of specialist staff, lack of training, supervision and clinician confidence, long waiting times, and high thresholds for referral to child and adolescent mental health services (CAMHS), as well as the burden and cost of travel and time needed to attend face-to-face treatment sessions (Radez et al., 2021).

Digital health interventions can address some of these barriers, potentially resulting in increased access to treatment. Technology is being adopted in clinical settings in the assessment and treatment of young people's mental health, and increasing evidence on its acceptability has resulted in its recommendation in international guidelines for some disorders, including for the treatment of depression (Chen et al., 2025). Digital health interventions for PTSD, including internet-delivered cognitive therapy for post-traumatic stress disorder (iCT-PTSD), have been developed and found to be effective and acceptable in adult populations (Ehlers et al., 2023). Preliminary outcomes suggest the feasibility of digital intervention in young people for universal psychoeducation, prevention, and stepped care. However, there remains a gap in the development, delivery, and evaluation of internet-delivered therapy for PTSD in young people (Schulte et al., 2024).

This study forms part of the Online PTSD Treatment for Young People & Carers (OPTYC) trial, an early-stage RCT of iCT-PTSD-YP (Smith et al., 2025). Qualitative methods are used to consider the lived experience of young people who took part in the trial and to explore the acceptability of iCT-PTSD-YP from their perspective. The study aims to generate a deeper understanding of the perspectives of young people by providing rich, participant-driven data to explore issues related to acceptability of digital delivery of therapy (Bevan Jones et al., 2020).

## 2. Method

### 2.1. Ethical considerations

Ethical approval was received from a UK Health Research Authority (HRA) Research Ethics Committee (REC; 19/LO/1354). The study is sponsored jointly by King's College London and South London and Maudsley Research and Development Office. The trial was preregistered (ISRCTN16876240).

### 2.2. Design

Semi-structured interviews were completed online with *n* = 20 young people recruited from both arms of the OPTYC trial. A topic guide was developed and reviewed by patient and public representatives (Appendix A).

### 2.3. Inclusion criteria

The main inclusion criteria for the OPTYC RCT were young people (aged 12–17 years) who had experienced a single-incident trauma and developed PTSD, as defined in the Diagnostic and Statistical Manual of Mental Disorders, Fifth Edition (DSM-5) (American Psychiatric Association, 2013). Full inclusion and exclusion criteria are available in Smith et al. (2025).

### 2.4. Participants

Thirty-one young people took part in the RCT. Of these, 30 were offered an interview and 20 were interviewed. Two declined an interview, eight did not respond to the offer of an interview, and one was not available for interview. Additional adherence metrics are reported in Smith et al. (2025).

In line with reflexive thematic analysis (RTA) principles, data saturation was not determined by sample size. However, based on guidance from a systematic review by Hennink and Kaiser (2022), it is expected that saturation would have been reached by approximately nine to 17 interviews within this study population.

### 2.5. iCT-PTSD-YP

The intervention delivers all components of manualized face-to-face CT-PTSD for young people (Smith et al., 2010), delivered online via a web app and supported by a therapist. The content is based on the theoretical underpinnings and core components of cognitive therapy for PTSD (Ehlers & Clark, 2000; Meiser-Stedman, 2002). The development and content of iCT-PTSD-YP are described in more detail in Smith et al. (2025).

Treatment components are delivered across 11 core modules. Eleven optional modules are also available to be used, based on the cognitive formulation and comorbidity (Table 1).

**Table 1.** Core modules and optional modules.

Core modules	Optional modules
What is PTSD	Guilt
Reclaiming life	Shame
It’s understandable	Anger
Your story	Physical difference
Hotspots	Rumination
Updating your story	Self-critical thinking
Triggers	Images
Sense of danger	Grief
Viewing the site virtually	Panic
Viewing the site in person	Sleep
Blueprint	Relaxation

Module content includes mood and PTSD symptom tracking questionnaires, psychoeducation presented through text and narrated animations, audio case examples, individual inputting into the app (e.g. activity schedules in ‘Reclaiming life’, uploading media to ‘Reclaiming life’, writing about the traumatic event in ‘Your story’, identifying problematic cognitions in ‘Hotspots’), and video testimonies of a young person talking about their experience of CT-PTSD.

Audio case examples and narrated animations were developed to guide treatment completion, video testimonies to boost motivation and engagement, media uploading to support with grounding and working with triggers, and mood and symptom trackers to guide self-monitoring and further support engagement. There is choice around an individual’s interaction with the app, including location of use, device used, timing and length of use, and mode of input (voice or written text).

App use can be individualized based on presenting problem, engagement, and motivation. Modules are released by the therapist as required, and accessed by the young person at any time once they have been released. Modules can be completed in one sitting or over several shorter sittings, with no prescribed length of time to be spent on modules. Core modules are released sequentially, generally following the order shown in Table 1, but optional modules may be released early if judged by the therapist to be helpful.

Therapists monitor progress, input into the app, and respond to questions. Young people and therapists can communicate via messaging on the app and SMS. Therapists also provide support through video or audio calls, the frequency and length of which are mutually agreed, based on clinical judgement and the young person's capacity, but usually once a week for 30 min. Therapist support aims to encourage log-in and use of the app, provide support with use, discuss barriers and any difficulties encountered, and implement treatment components.

### 2.6. Procedure

Interviews were conducted remotely and video recorded using Microsoft Teams between November 2021 and December 2022. Interviews lasted between 10 and 55 min (average length 31 min). Of the 20 young people interviewed, 10 had been randomized to receive treatment straight away and 10 after 16 weeks on a waiting list. Of these, 19 had completed treatment and one did not receive treatment owing to natural recovery while on the waiting list; however, the latter was interviewed about their experience of participating in the trial. Interviews were conducted by three members of the study team. All interviewers were female, white British, and educated to master's level. Interviewers did not interview anyone whose assessment or treatment they had been involved in. Interviews were transcribed verbatim and imported into NVivo 14 (Lumivero, 2023) for analysis.

### 2.7. Analysis

RTA was used to analyse the data. This approach was chosen for this dataset owing to its recognition as a robust, systematic framework used to identify, analyse, organize, describe, and report themes that are found within a dataset in relation to the research question (Braun et al., 2024), its flexible methodology, which allows a rich and detailed interpretation of complex data, and its frequent use in psychological research and increasing use in RCTs to facilitate the interpretation of trial results (Hardy & Llewelyn, 2015). Its consideration of multiple perspectives, ability to highlight similarities and differences between participants, capacity for the generation of unanticipated insights, and recognition of the researcher's subjectivity as being integral to the process make RTA a highly appropriate method of analysis for this dataset and explorative research design.

A critical realist epistemological stance to data analysis was taken (Wiltshire & Ronkainen, 2021). This approach recognizes that there are multiple realities but with some common features. The ontological assumption of this stance is that although reality is shifting and susceptible to many interpretations, meanings, and values, there can be shared qualities of human experiences. The epistemological assumption is that claims of truth can be evaluated in terms of real-world evidence and therefore some measure of shared understanding is possible. We can therefore obtain some measure of shared understanding of the participants’ experiences by examining and evaluating their unique perspectives, while also recognizing that they are susceptible to many interpretations and meanings. Owing to the exploratory nature of the study, the analysis used an inductive thematic approach and therefore was not shaped by existing theory but, rather, was generated from the data in a bottom–up approach that prioritized the interpretation of the young people's experiences. Data were coded at both the semantic and latent level, allowing consideration of both the meaning that was made and communicated by the young person and the interpretation of this by the researcher. Throughout the interviews and the analysis, reflections were made in a journal to ensure reflexivity and awareness of the researcher's epistemological standpoint.

### 2.8. Reflexivity statement

A central component of RTA is that the position of the researcher and the contributions that they make are necessary and important parts of the process and provide a valuable tool to be drawn on in the analysis. Throughout the process, the researchers reflected on their position, the position of their supervisors and their positioning in relation to this, and the beliefs that they held and how these may influence the process. The lead researcher reflected on her position as a white British female who held two professional roles during the study: the first as a research assistant involved in the development of study materials, recruitment, treatment, and data collection; and the second as a doctoral student involved in the further collection and analysis of data.

Continuous reflexivity through discussion with supervisors and reflexive journalling was helpful for the researchers to reflect and draw upon their knowledge, beliefs, and experiences. This included reflection on their alignment with the research trial, background in cognitive behavioural therapy (CBT), the cognitive model of PTSD and its theoretical underpinnings, their knowledge and experience of using CT-PTSD in clinical practice, and their involvement in the development of iCT-PTSD. This allowed reflection on the influence that these positions may have had on the development of questions, questioning style, interactions with young people, the potential for social desirability or demand characteristic effects occurring during the interviews and on their engagement with the data, data interpretation, and the generation and presentation of themes. This process was also helpful in addressing potential study limitations.

### 2.9. Procedure

The process of analysis followed guidelines presented by Braun et al. (2024) (Table 2).

**Table 2.** Process of analysis.

Phase	Process
Familiarization with the data	Video-recordings were listened to, and the data were transcribed verbatim. This was completed by both the lead researcher and another researcher in the OPTYC team (JS). Transcripts were subsequently read multiple times by the lead researcher (HG), who took initial notes during this process to increase familiarity with the data.
Generation of initial codes	Initial codes were generated using line-by-line coding of the transcripts. This was done by the lead researcher using NVIVO 14 software. This created a collated group of extracts from the transcripts which underpinned the initial codes and therefore ensured that the codes were reflective of the data. To increase reliability, the initial codes were discussed in a thorough consultation process with two senior members of the OPTYC team (PS, RMS).
Generating themes	Themes were generated by reviewing the list of codes identified in the transcripts. Codes were grouped together, and a list of potential candidate themes and subthemes was produced. The themes were then presented to two senior members of the study team (PS, RMS) to discuss the interpretation of the codes and decide on a potential list of themes.
Reviewing potential themes	The candidate themes and subthemes were reviewed and refined through further discussion with senior members of the team (PS, RMS). The candidate themes and subthemes, alongside supporting extracts from the data, were individually discussed and scrutinized by the research team until a consensus was achieved. This aimed to enhance reflexivity and validity in the process.
Defining and naming themes	Themes were finalized, defined, and named. A thematic map was created and reviewed by PS and RMS to agree on a consensus, finalize the candidate and subtheme definitions and names, and agree on the final format.
Production of the report	A write-up of the analysis was conducted, and involved writing, redefinition, and revision. Changes were documented and reflective notes were kept during this process. A report was produced that took into account the order of the themes and the need to present the themes logically and meaningfully. This aimed to create a clear narrative that reflected the data. The report was reviewed by the study team and, once completed, the lead researcher and supervisors conducted a final inspection of the report.

## 3. Results

### 3.1. Demographics

Demographic information is presented in Table 3.

**Table 3.** Characteristics of the participants.

Participant pseudonym	Age (years)	Gender	Ethnicity	Trauma type
Ruby	15	Female	White British	Witnessing violence towards family member
Rose	17	Female	White British	Sexual assault or rape
Lucy	15	Female	White British	Transport accident
Max	15	Male	White British	Physical assault
Anika	16	Female	White British	Sexual assault or rape
Immy	13	Female	White British	Medical procedure
Niamh	15	Female	White and Black Caribbean	Sexual assault or rape
Frankie	16	Female	White British	Sexual assault or rape
Nichole	16	Female	White British	Sexual assault or rape
Hayley	16	Female	White and Black Caribbean	Physical assault
Charlotte	14	Female	Black British	Robbed by threat, force, or weapon
Anna	17	Female	Black British	Sexual assault or rape
Hannah	14	Female	White Other	Transport accident
Kiera	15	Female	White British	Sexual assault or rape
Bella	16	Female	White British	Family member dying
Samuel	15	Male	White Other	Robbed by threat, force, or weapon
Cora	16	Female	White British	Witnessing family member injured
Katie	14	Female	Black British	Sexual assault or rape
Taylor	13	Female	White British	Witnessing violence towards family member
Grace	16	Female	White British	Family member dying

### 3.2. Analysis

Three overarching themes were generated: (1) Accessibility, (2) Engagement, and (3) Expectations and outcomes. Themes, subthemes, and example quotations are presented to illustrate the findings. Table 4 presents an overview of themes and subthemes.

**Table 4.** Overarching themes and subthemes.

Overarching theme	Subtheme	Example quotation
Accessibility	The app makes treatment accessible and available	‘Everything that’s been thought of you have access to, you know, you can speak to your therapist on there, there’s videos, there’s explanations. I think it’s all round a nice place to have everything in one place. And then you just click on it, click out and yeah, I think it’s a really good thing that’s been made.’ (Frankie)
	Internet-delivered therapy is flexible and convenient	‘I didn’t like the idea when I did face-to-face therapy of having to, like, physically take time out of my day to go to a room and talk to someone, like, that just annoyed me after a bit. So, with this, like, if I was sitting in bed and I thought I have time now, I get my computer and just do it in bed with my pyjamas on, it was very flexible and like, if I wasn’t feeling good, I would just stop or I would just wait. That’s why I didn’t get on with face-to-face. Like, I compared it a lot with my old therapy, and I like this one more. It had a lot more benefits and advantages.’ (Katie)
Engagement	Therapist support is important	‘I do think I need that kind of contact whether it’s like over the phone or in person with a therapist. I think that was definitely something that was vital for me and like I don’t know how helpful it would have been without the calls as well.’ (Cora)
	You need to be autonomous and self-motivated, and certain things can make it harder	‘I struggle with motivation all round, but I then always would try and tell myself that this would help me feel better in the long term and I would remember, that I would remember these techniques for the rest of my life, even if I didn’t want to. So, it was worth, you know, sitting down for 20 min to finish some work rather than lying in bed.’ (Kiera)
	The app is engaging, but technical issues can get in the way	‘I think it was quite like unique and I feel like it was enough to make me want to be on it because it looks nice if that make sense.’ (Hayley) ‘Just like the tech problems really, like if it didn’t work or like teams went down or the wifi played up or something we couldn’t really do much.’ (Lucy)
	It’s important to feel safe and comfortable	‘If I was not feeling so good after, I could just kind of be in my room, in my … it’s ‘cause it’s like a safe space, I guess. I like being in my room with my cat and it calms me down a bit, so yeah, I think I like that I could be in my, like, a safe place.’ (Anika)
Expectations and outcomes	Not knowing what to expect, how it would work, or if it would help – a new experience, during a new time	‘During the first few weeks, I was just like what can an app do?’ (Max)
	There are specific techniques that help the most	‘I think the most helpful bit for me was writing out my story and then putting in my here and now, because that’s something I find myself keep going back to when I’m struggling or having a bad day and I know I’m not speaking to my therapist for a few days. I can just log on and read that and that does really help me.’ (Rose)
	It was hard work, but it helped	‘I found the more I did the treatment, the more I realized how helpful the app actually was. I didn’t give it as much time as I should have at the start, but I think it was actually really helpful. So, I would say spend more time on the app than you might realize or might think you need to because there’s quite a lot in there to benefit from.’ (Samuel)

#### 3.2.1. Theme 1: Accessibility

This theme covers the perception of iCT-PTSD-YP as a means of increasing treatment accessibility. Within this theme, two subthemes emerged.

##### 3.2.1.1. Subtheme: The app makes treatment accessible and available

The app’s availability and usability, including its ease of access, use, and navigation, as well as its clearly outlined therapeutic content, were perceived as providing access to consistent and readily accessible treatment.

This appeared to provide the opportunity to adopt a more self-guided approach to treatment.

It's available whenever you need it … I wasn't forced to go too quick, and I still have access, so it's like I could revisit all the modules whenever I wanted and I could use the information that I had come across before to easily like, like help me figure out what I wanted to put in the next module. (Anna)

This was also viewed as being developmentally appropriate, beneficial to those who may find traditional talking therapies challenging, providing support with addressing cognitive and emotional avoidance, and aligning with young people’s preferred ways of communicating.

It's a good way to do that sort of therapy with teenagers. Especially because obviously most teenagers are always on their phones or like computers, so it was a cooler way of doing it than sort of sitting in a room talking to someone face-to-face. And I know, especially because of the nature of the reason I have had therapy, sometimes, I find it hard to get my words out and express the way I’m feeling. So, I think as a generation of younger people we find it easier to just type how we are feeling and get it all out that way. (Rose)

Young people also shared their experiences in comparison to CAMHS, stressing the length of waiting lists as a barrier to access to assessment and treatment. Being part of a trial receiving iCT-PTSD was, therefore, perceived as a novel way of increasing access to more timely and thorough assessment and evidence-based treatment.

I think this was the quickest I’ve ever waited for treatment ever! (Hayley)

##### 3.2.1.2. Subtheme: Internet-delivered therapy is flexible and convenient

The flexibility and convenience of iCT-PTSD-YP appeared to address accessibility issues such as time, location, and travel burdens.

It was nice like being able to do it after school because like I’ve done things before, like I’ve done CBT before, and it was always, like, I would have to leave school to go to an appointment. So, it was definitely much more accessible than any that I had before. (Cora)

The therapy was perceived to provide flexibility in treatment, allowing participants to go at their own pace, being adaptable to individual preference and providing support with emotion regulation.

This was beneficial to young people who needed to fit therapy around other commitments, including school, exams, and extracurricular activities.

Being able to do it from home helped a lot around revision. (Nichole)

I was doing a school play at the same time, which I was advised to do, so it was quite nice to be able to do that for my enjoyment … but it was really nice to be able to do things on my own terms and still manage to have the calls with my therapist. (Grace)

#### 3.2.2. Theme 2: Engagement

This theme portrays young people's experiences of engaging with iCT-PTSD-YP. Within this theme, four subthemes were produced. Important findings emerged throughout the subthemes that require consideration when supporting young people to engage with iCT-PTSD. These include the need to recognize important barriers to engagement, including the level of time and commitment required, level of autonomy needed, and awareness of individual and contextual factors that may act as potential barriers to engagement.

##### 3.2.2.1. Subtheme: Therapist support is important

Therapist support was found to be an important element of the experience of iCT-PTSD-YP, enhancing retention and engagement. Certain therapist qualities were identified as being helpful, including therapist availability and flexibility, the amount of support offered, type of contact, timing of sessions, and working around individual schedules. This contributed to the participants’ feeling of having their time valued, the treatment appearing personalized, and the impression of being well supported.

There was always an option to like to do it with some help or like by yourself, so you just had like the freedom but also, like, I just knew that I was sort of supported. In a way, they made sure that you knew that their time revolves around like your time and availability. (Bella)

Young people found therapist contact through calls and mid-week texts helpful for motivation and engagement.

I think having the check-in calls was a helpful kind of point of discussion because it would give me kind of the week to do the app work and then we’d discuss what I’d done on the app, so I knew having that to work forward, too, was something that would keep me on my toes basically and keep me going. (Frankie)

A personalized and tailored approach was viewed as helpful for engagement. This included therapy being individualized and the therapist offering support outside the app content.

They took the time to get to know me … like, spoke to me about personal stuff as well and were very interested in my life outside of just therapy … and it made me like enjoy it more, and the way they were, made me a lot more into it than I would have been if I didn't really like the person. (Taylor)

Although the remote nature of the therapist contact was seen as useful for engagement and preferable to face-to-face treatment, a minority of participants suggested the potential for a hybrid approach. This was described as meeting with their therapist either at the beginning of treatment or during more difficult parts of treatment, or to mark the ending, and may have further supported their engagement.

I think there are aspects of the treatment where I would have preferred face-to-face treatment … I think the story and guilt. (Kiera)

I think maybe meet for one session. (Charlotte)

##### 3.2.2.2. Subtheme: You need to be autonomous and self-motivated, and certain things can make it harder

Self-motivation and autonomy were found to be needed for iCT-PTSD. For some young people this was difficult, whereas for others this presented as a means of taking ownership over treatment, which appeared to provide support with motivation and engagement. Young people spoke about individual factors that could impact on engagement. These included managing the time commitment around other life activities.

Sometimes, where it was just a call and not in person, it was easy to forget … and uhm, sometimes, it was hard to complete them in a week just because, yeah, sometimes other things would get in the way and stuff like that, and I couldn't get round to it. (Bella)

Variation in mood appeared to have an impact on engagement with treatment for some young people. This included the impact of low mood on the ability to engage with iCT-PTSD-YP and the temporary impact of the treatment modules on mood.

I think sometimes having low moods made me not want to do it or like sometimes after a module I would feel really drained. (Charlotte)

Sometimes in like the treatment stuff, like with the story and stuff, it kind of made me feel a bit worse, so I was like, I think this is the reason and I was like, why am I doing this and stuff like that, like, I don't want it to make me feel worse again. (Ruby)

However, seeing positive changes appeared to contribute to the level of motivation to continue.

Yeah, like, I felt like I always have a bit of trouble with motivation, but like, sometimes it was really hard to really get myself to do it. But like at the end of it, I always realized it was better to have done it than to have left it, like, I always end up, like, being grateful that I pushed myself to do it. (Katie)

##### 3.2.2.3. Subtheme: The app is engaging, but technical issues can get in the way

Young people found that aspects of the design and content of the app were engaging.

The use of media appeared to help with the memorability and generalizability of the techniques.

If I’m not going on the app, I will imagine the picture that I put on my here and now and it just helps me to feel a bit more kind of comfortable and less anxious. (Grace)

The video testimonies contributed to the normalization and validation of symptoms, and the animations were experienced as being useful for psychoeducation and enhancing the memorability of techniques. These features helped with engagement in different treatment components.

The voice recordings of like the different like people and their different experiences, and their like examples of what they would do because you would see three completely different events. You could kind of tie yourself with one of them, or a few of them. And then you feel like way less alone because you’re like, OK, cool like this is actually really normal, if that makes sense and I feel like with the cartoons it would just get into your head a bit more … I feel like people just like cartoons, so I think it's just a bit easier to explain things. (Hayley)

The app was experienced as facilitating engagement in the more difficult aspects of treatment, such as telling the story of their trauma. Young people valued the ability to have a choice regarding the mode of working on the story (written narrative or audio recording), the ability to edit their story, and therapists being able to edit it live on the call.

It helped seeing what I’ve written, you know, being able to access and go back and read it without having to rewrite it. (Samuel)

So like if you were having a hard time, like writing something down, you don't need to like force yourself to do it. You could just ask to yeah to get help that way. (Anna)

Despite finding the app engaging, occasional technical issues, including not being able to log on to the app, scheduled downtime, and technical glitches resulting in temporary missing data, were found to be a barrier, causing frustration and preventing completion even when motivation was present.

Yeah, sometimes you get out of school and you’re like I’m going to go do some productive work today. I’m going to go do my PTSD homework and then you’re like oh well I can't get on it so it's not gonna happen. (Kiera)

##### 3.2.2.4. Subtheme: It's important to feel safe and comfortable

Access to remote therapy was seen to promote a sense of safety and comfort, both in engaging with the therapeutic content and for managing difficult emotions afterwards.

I think it's like so much easier to, like, do it from home, particularly when it's something that is like quite like upsetting and like having to like, go over like, you’re, like, trauma being able to do that, like in the comfort of, like, your bed. Like, it's just nice. (Cora)

Being comfortable in their own environment appeared to encourage young people to feel safer to engage and communicate.

You can do it without camera, so you don't really have to matter what you look like. You can come home and sort of get comfortable. And I think because it's a home environment you feel more inclined to share personal details. (Bella)

#### 3.2.3. Theme 3: Expectations and outcomes

This theme captures young people's reflections on their expectations of receiving iCT-PTSD-YP, as well as their perceptions of its outcomes. Within this theme, three subthemes emerged.

##### 3.2.3.1. Subtheme: Not knowing what to expect, how it would work, or if it would help – a new experience, during a new time

There appeared to be some uncertainty about iCT-PTSD-YP, owing to both a lack of expectations about its efficacy and difficulty with imagining how their symptoms could change.

I definitely went into it very kind of sceptical about how I could, like, feel better, you know, because it had been like years of me feeling like that. (Cora)

The timing of the study, occurring during the coronavirus disease 2019 (COVID-19) pandemic, appeared to facilitate openness to try a new type of treatment, as other services were either not running or also running remotely.

I think [the pandemic] probably made it happen. I don't think I would have known about this treatment, the whole online and app work. I don't think it would have been a thing really for me at least before lockdown. So, it's made it really accessible and super convenient especially in such bizarre times where everything is online now, but it's actually really good. (Grace)

##### 3.2.3.2. Subtheme: There are specific techniques that help the most

Specific modules and techniques were identified as being the most helpful for symptom improvement. These included psychoeducation, grounding, and working on trauma memories. There was a broad consensus that writing a narrative and updating hotspots with what they learned in therapy (e.g. through cognitive restructuring) and changing unhelpful coping and cognitions through behavioural experiments were the most helpful techniques.

I just think, the skills I have learnt, the here and now was one of the most helpful things and like the um the safety, like the things I used to do to try and make myself feel safe and how I have managed to like, I don't look over my shoulder as much now and stuff yeah it's just the skills it's given me but my story has probably been the most helpful thing. (Rose)

##### 3.2.3.3. Subtheme: It was hard work, but it helped

The findings suggest that to gain the most from the treatment, young people were required to put in hard work and commitment, and to trust in the process. The treatment was found to be challenging but worth engaging with in terms of the outcomes.

But I think obviously that was part of the process, it's going through the bad so I can get to the good and the better. I feel like my PTSD like it's got, it's really like, gotten better or like I can do stuff that I wouldn't be able to before. (Hannah)

Overall, young people appeared to find the treatment acceptable, and reflected positively on their outcomes while recognizing the challenges and hard work that it required. The majority of young people perceived that they had made some improvements in their levels of PTSD symptoms, which were reflected in their scores on the questionnaires on the app, which, in turn, was seen to have impacted positively on their lives.

## 4. Discussion

This study explored the experiences of young people receiving therapist-supported iCT-PTSD-YP as part of an RCT. Interview data were analysed using RTA, and three overarching themes were generated.

The therapist-supported digital therapy was perceived as accessible, available, and easy to use and navigate; iCT-PTSD-YP was found to be convenient and flexible; and taking part in an RCT offered the opportunity to access thorough assessment and treatment. Level of engagement depended on individual and contextual factors and was helped by the support of a therapist, a well-designed digital platform with engaging therapeutic content, and receiving treatment in a safe and comfortable environment. iCT-PTSD-YP was perceived as efficacious by the majority of young people, but requiring a level of autonomy, hard work, and commitment to treatment.

### 4.1. Findings in context

An accessible and usable app was perceived to provide structure to the treatment. This mirrors findings from research on face-to-face TF-CBT, where the provision of well-organized, structured, and sensitively paced sessions was found to enhance engagement (Neelakantan et al., 2019). Therapist-supported apps may, therefore, have the potential to provide additional structure and containment for young people and therapists. This may be beneficial for therapists who find working with trauma-exposed young people difficult and demanding (Allen & Johnson, 2012), and may increase therapists’ confidence by challenging therapist avoidance due to concerns about increasing distress and retraumatization (Finch et al., 2020).

In general, iCT-PTSD-YP was found to be acceptable and feasible. This finding offers a positive position on the acceptability of remote treatments (Hollis et al., 2017). Young people in this study did not have a preference for a full course of face-to-face therapy, and reported benefits of both using an app and having remote sessions with a therapist.

This study supports a different perspective from findings that suggest that young people may perceive remote therapy as less individualized and providing less therapist contact (Wisman et al., 2023), by providing evidence that iCT-PTSD can feel personalized and that remote contact with a therapist offers additional benefits of flexibility and the impression of constant availability of the therapist, even if overall contact time is reduced, as it was here (Smith et al., 2025). Alongside other research (Leigh et al., 2023), these findings suggest that therapist-supported app-based treatments may offer a unique and different experience of remote treatment, with the potential to enhance the support felt by participants during therapy.

Young people found that they could feel safe, comfortable, and contained when engaging with iCT-PTSD-YP. Different elements of safety and stability, including psychological, physical, and relational safety, are important factors that should be established when working with trauma. Young people who have received face-to-face treatment recognize that components of safety are particularly important when engaging with TF-CBT (Neelakantan et al., 2019). It is therefore an encouraging finding that these components can be achieved with iCT-PTSD-YP.

A therapist who was supportive, receptive, and flexible, as well as providing valuable guidance and encouragement, was valued. These findings correspond with previous research on digital health interventions, which found that therapists who adopted flexible communication helped to motivate young people to engage with therapy (Leigh et al., 2023). The results also support findings that therapist support is an important factor associated with satisfaction and adherence (Lehtimaki et al., 2021; Smart et al., 2023). In agreement with qualitative findings from young people who have received face-to-face TF-CBT (Neelakantan et al., 2019), the findings from this study provide evidence that safety within the therapeutic relationship is an essential component of engagement in internet-delivered therapies.

Overall, the most valued components of treatment were consistent with the current literature base. Relative to efficacy studies of different aspects of PTSD treatment, psychoeducation, writing a written narrative, and updating hotspots with learning from therapy and cognitive restructuring were perceived to be the most helpful parts of treatment. An essential component of CT-PTSD involves the construction of a trauma narrative (Smith et al., 2010). This study provides further evidence in support of this in both CT-PTSD and iCT-PTSD, and adds to the qualitative literature base that shows that young people also find trauma-exposure work and trauma narrative activities helpful (Dittmann & Jensen, 2014; Salloum et al., 2015).

Young people who received face-to-face TF-CBT have suggested that technology may be harnessed to improve the appeal and age-appropriateness of therapeutic content (Neelakantan et al., 2019). The current findings provide evidence of value in the use of media for engaging in the content and memorability of techniques. The use of an app therefore may provide a means of providing more appealing and engaging therapeutic content for young people.

A promising finding from the RCT was the substantial reduction in self-reported symptoms (Smith et al., 2025). This matches literature on experiences of face-to-face TF-CBT, which finds that, despite initial reservations and hesitancy, treatment is perceived as helpful and resulting in improvement in a range of cognitive, emotional, behavioural, and interpersonal areas (Neelakantan et al., 2019).

### 4.2. Limitations

The design of the study is robust, using rigorous qualitative methodology. While there were different perspectives, the themes were mostly consistent across all young people.

Limitations of the study include the somewhat homogeneous sample of predominantly female participants, aged 15/16, and of white ethnicity. This raises wider questions around accessibility and generalizability, as the sample may not be representative of the young people under CAMHS.

Eleven young people who took part in the OPTYC trial did not complete an interview. It is therefore possible that the perspectives captured may be different from those of the people who did not complete interviews.

Interviewers were either former or current trial staff members, which introduces the potential for social desirability or demand characteristic effects to occur during the interviews. This was mitigated by ensuring that, if an individual interviewer had conducted a research assessment with a young person or been involved in their treatment as part of the trial, they did not subsequently conduct the interview with that young person.

The analysis was carried out by a researcher who had been involved in aspects of the wider trial, including the development of trial materials, recruitment, assessment, and treatment. This introduces the possibility for bias in the completion of the interviews and analysis owing to the researcher having prior knowledge of the treatment and preliminary outcomes, as well as her professional positioning, which may yield interpretations that align with hopes for positive reflections on trial outcomes.

Despite the interview questions being designed to invite constructive feedback and identify potential criticisms of iCT-PTSD, the findings that emerged were generally more positive in their stance on the intervention, with less critical material emerging within the themes. This could suggest the presence of social desirability and demand characteristics effects occurring during the interviews and the potential for interpretive bias. This was addressed through ongoing reflexivity, with researchers critically considering their positions throughout the analysis and balancing this with what emerged from the data. This process included reflecting on the strengths in the development of the treatment, which used co-design principles to involve young people in developing the therapy app and website (Bevan Jones et al., 2020; Smith et al., 2025) and may have contributed to the positive outcomes.

In keeping with RTA principles, the researchers were careful to consider their stances, which were used as a tool in the analytic process through the use of reflective journalling and discussion. This helped to directly address any analysis or interpretation that may be based solely on prior knowledge and their professional positioning of alignment with the trial and belief in the treatment.

### 4.3. Future considerations

In future, it will be important to capture the perceptions of a wider range of young people, from more diverse backgrounds and with a wider range of experiences, including those who have chosen to discontinue online treatments. It is also important to separate the findings of studies looking at general remotely delivered treatment, stand-alone apps, and therapist-supported apps.

This study was carried out during the COVID-19 pandemic. During this time, young people experienced many aspects of their life being transferred to online means, and therefore further research is necessary to understand their perspectives in a post-pandemic world.

## 5. Conclusion

This study provides preliminary evidence that iCT-PTSD-YP is feasible and acceptable to young people. However, it highlights key considerations that need to be addressed to improve acceptability as well as engagement. This includes thinking about individual, internal, and contextual factors, and improving the overall functioning of the app.

## Data Availability

The data are not publicly available owing to privacy or ethical restrictions.

## Appendix A

*Topic Guide*

**OPTYC: Your Experience of PTSD Therapy**

**CODE:** ________________________

**DATE:** ________________________

**INTERVIEWER:** ________________________

### 1. Overall experience of treatment

1. Overall, how satisfied were you with your treatment?
  Please rate from 0–5: Not at all satisfied 0 1 2 3 4 5 Very satisfied
2. How supported did you feel during treatment?
  Please rate from 0–5: Not at all supported 0 1 2 3 4 5 Very well supported
3. How convenient did you find completing your sessions each week?
4. In general, what did you find helpful during your treatment?
5. What do you remember best about treatment?
6. What did you like most about treatment?
7. What techniques did you find most helpful?
8. Were there parts of treatment that were not helpful?
9. What did you like least about treatment?
10. What techniques were not helpful?
11. Was there anything in therapy that made you feel worse?

### 2. The app

12. What did you think when you were allocated to PATH online treatment?
13. What did you think of the PATH app after doing the first module?
14. Looking back, what do you think of the app in general?
15. What were the best things about the app?
16. What did you like least about the app?
17. What did you think of the design and layout of the app?
18. How could the app be improved?
19. I would recommend the ICT therapy to a friend who is experiencing similar difficulties to me.
  Please rate from 0–5: strongly disagree 0 1 2 3 4 5 strongly agree
20. For you, what were the benefits or advantages of online therapy?
21. For you, what were the disadvantages or challenges of online therapy?

### 3. Your therapist

22. Was it helpful to talk to your therapist on the phone?
23. Would you have liked to have more contact with your therapist? Less?
24. Would you have preferred to come to the clinic to see your therapist face-to-face?
25. Did your therapist do or say things that were especially helpful?
26. Did your therapist do or say anything that you didn't find helpful?

### 4. Motivation

27. Did you have any problems in motivating yourself during treatment?
28. What helped you stay motivated?

### 5. Time spent

29. How much time do you think you spent on your treatment each week, including time spent outside the programme?
30. Looking back, what do you think is the ideal amount of time young people should spend on their treatment each week, including time spent outside of the programme?

### 6. COVID-19 impact

31. What impact do you think the COVID-19 pandemic has had on your treatment?
32. Outside of the COVID-19 pandemic, do you think you would have participated in this treatment?

### 7. Experiences of being part of the OPTYC trial

33. What was your experience of being in the trial?
34. What was your experience of being randomised?
35. Did the randomisation process impact your decision to take part in the study?
36. What was your experience of the assessment?
37. What was your experience of completing the questionnaires at your assessments?
38. Was the information given and explanation of OPTYC study understandable/sufficient?
39. What did you think of the wait time for treatment?
40. Is there anything you’d recommend to be different if we were to run a study like this again?
41. Do you have any other comments about your experience of participating in the OPTYC Study?
